# Assessing Quality of Life and Associated Factors Among Caregivers of Children With Chronic Conditions at Tamale Teaching Hospital: A Cross‐Sectional Study

**DOI:** 10.1002/hsr2.73318

**Published:** 2026-09-30

**Authors:** Rhubamatu Iddrisu, Abdul Rauf Alhassan, Mariam Kaleem‐Ahmed, Yaa Nyarko Adjeso

**Affiliations:** ^1^ Nurses and Midwives Training College Tamale Ghana; ^2^ Department of Surgery Tamale Teaching Hospital Tamale Ghana; ^3^ School of Kinesiology and Health, Queen's University Kingston Ontario Canada; ^4^ Ghana Organization for Maternal and Child Health Tamale Ghana; ^5^ Biology Department Oglethorpe University Atlanta Georgia USA; ^6^ Department of Population and Reproductive Health, School of Public Health University for Development Studies Tamale Ghana

**Keywords:** caregivers, children, chronic illness, Ghana, quality of life, WHOQOL‐BREF

## Abstract

**Background and Aim:**

Children with chronic illnesses are particularly vulnerable, they often face long and frequent hospitalizations that impair their physical, psychological, social, and educational well‐being. Hospital stays also affect the quality of life (QoL) of their caregivers, who may experience stress, anxiety, depression, and financial difficulties. This study aimed to assess the QoL of the caregiver of a chronically ill child in Tamale Teaching Hospital.

**Methods:**

A descriptive cross‐sectional design using quantitative data was used for this study in Tamale Teaching Hospital. Descriptive and inferential analysis were done. The significance was set at *p* < 0.05.

**Results:**

The mean general QoL score (single‐item measure) was 32.6 ± 24.0, indicating poor perceived overall life quality. In contrast, the overall QoL score derived from WHOQOL‐BREF domains was 49.9 ± 13.6, indicating a moderate level of QoL. Domain‐specific mean scores were 47.6 ± 17.0 (physical), 50.8 ± 14.7 (psychological), 53.0 ± 19.1 (social), and 47.9 ± 14.4 (environmental). Overall, 50.6% (45/89) of caregivers had good overall QoL. Factors significantly associated with QoL included type of chronic condition, duration of admission, caregiver education, occupation, and religion (*p* < 0.05). In multivariable analysis, unemployment was associated with lower odds of good QoL (AOR = 0.27, 95% CI: 0.08–0.88), while caregivers of children with sickle cell disease (AOR = 7.75, 95% CI: 1.23–48.90) and those of Islamic faith (AOR = 4.05, 95% CI: 1.24–13.24) had higher odds of good QoL.

**Conclusion:**

Caregiver QoL was moderate and associated employment status, religion, and the child's illness type. Support for vulnerable caregiver groups is essential to improve their well‐being.

## 1 Introduction

Chronic illnesses are long‐term health conditions that require ongoing medical care and management [1]. They affect millions of people worldwide and pose significant challenges for patients, families, and health systems [2]. Among the most vulnerable populations are children with chronic illnesses, who often experience prolonged and frequent hospitalizations as part of their treatment [3].

Chronic illnesses (CI) are on the rise and also pose significant challenges to children's physical and social well‐being [4]. According to autopsy data from the Korle‐Bu Teaching Hospital, the largest tertiary care center in Ghana, non‐communicable diseases (NCDs) account for 41% of adolescent mortality in the country [5]. Moreover, anecdotal evidence from pediatric admissions for 2021 and 2022 indicated that chronic conditions among children is on rise Tamale Teaching Hospital.

Long‐term hospitalizations can have a significant negative effect on the lives of those who are chronically ill [6]. These children perceive hospitalization as a continuous, frequent event rather than merely a short inconvenience. These hospital stays can have detrimental effects on their physical, psychological, and social development, as well as their quality of life (QoL) [7]. The consequences are extensive, affecting all facets of their lives, for instance, hospitalization can disrupt their normal routines and activities, limit their opportunities for socialization and education, and expose them to stress and trauma [8].

Moreover, chronic illnesses could have a detrimental impact on the overall QoL of those who experience prolonged hospital stays. They may face higher levels of stress, anxiety, and depression due to the physical pain, uncertainty, and fear associated with their health condition [9]. Long‐term hospitalization can also make a person lose their sense of independence and control, which could make them feel powerless over their own lives [10].

Chronic illnesses not only affect the patients, but also their close family and caregivers who face difficulties and burdens due to their caregiving role [11]. Family caregivers provide vital physical, emotional, social, and economic support to chronically ill patients, but they often overlook their own health and well‐being [12]. As they deal with the demands and stressors of caregiving, they may experience burnout symptoms, such as fatigue, anxiety, depression, and isolation [13]. Caregiving can also have an impact on their financial situation, social relationships, and personal goals [14]. This can further complicate the care for the patient and create a vicious cycle.

However, based on the current research and to the best of the researcher's knowledge, there limited research on the specific impact of hospitalization on caregivers in Ghana, especially in Tamale, where the health system faces many challenges and resources are limited. Therefore, this study also aims to fill this gap by assessing the quality‐of‐life caregivers of the chronically ill children at TTH.

## 2 Methodology

### 2.1 Study Design and Setting

This study adopted a descriptive cross‐sectional design with quantitative data at the pediatric unit of Tamale Teaching Hospital. This consist of pediatric ward, the children's emergency ward (CEW) and the specialist clinics.

### 2.2 Study Population

In this study, the population consists of all children with chronic diseases and their immediate family members who seek care at the Tamale Teaching Hospital in Ghana. The estimated number of children seeking care for various chronic medical diagnosis at the pediatric clinics is 101.

### 2.3 Sample Size Calculation and Sampling Procedure

The Yamane sample size calculation formula was used to determine the sample size for quantitative data. It takes into account the population size (*N* = 100), and the level of precision (e = 0.05).

Therefore:

$$n=\frac{N}{1+N({e)}^{2}}\;n=\frac{101}{1+101({0.05)}^{2}}=80.6.$$

For non‐response 5% of the calculated sample size was added (10% of 80.6 = 8.1), thus 80.6 + 8.1 = 88.7 or 89, hence a total of 89 respondents were to be sampled for this study analysis using simple random sampling approach.

A sampling frame was created from the list of caregivers attending the pediatric unit during the study period. Eligible participants were assigned identification numbers, and simple random sampling was conducted using a random number generator to select participants.

### 2.4 Inclusion and Exclusion Criteria

The caregivers and children with chronic diseases seeking care at Tamale Teaching Hospital were included in this study. Those excluded were caregivers of children with chronic diseases, newly diagnosed and very sick children on admission. Chronic conditions were defined as illnesses lasting ≥ 3 months requiring ongoing medical care. “Very sick children” were defined as those requiring emergency or critical care and whose caregivers were unable to participate in interviews.

### 2.5 Data Collection Tool

The survey questionnaire (WHO BRIEF‐26) was used to collect data on the QoL of the careivers of children with chronic diseases. The WHOQOL‐BREF instrument consists of 26 items grouped into four domains: physical health, psychological health, social relationships, and environmental health. Each item is scored on a 5‐point Likert scale. Negatively phrased items were reverse‐coded according to WHO guidelines. Raw domain scores were calculated by summing item scores within each domain and then transformed to a 0–100 scale following the WHOQOL‐BREF scoring manual, where higher scores indicate better QoL. In cases of missing data, domain scores were calculated if at least 80% of items within the domain were completed, as recommended by WHO guidelines.

The categorization of QoL scores was put into poor (≤ 45), moderate (46–64), and high (> 65) was based on previous studies conducted in similar settings [15]. However, it is acknowledged that WHOQOL‐BREF does not provide universally standardized cut‐off thresholds, and these categories were used for descriptive and comparative purposes only.

Operational definitions

History of long admission was defined as hospitalization lasting ≥ 14 days within the past 6 months.

### 2.6 Data Analysis

Descriptive statistics were used to summarize the data. Continuous variables were assessed for normality using appropriate methods. Normally distributed data were summarized using mean ± standard deviation (SD), while non‐normally distributed data were summarized using median and interquartile range (IQR). Chi‐square tests were used to assess associations between categorical variables. Logistic regression analysis was performed to identify predictors of QoL. Adjusted odds ratios (AORs) with 95% confidence intervals (CIs) were reported. All tests were two‐sided, and a *p*‐value < 0.05 was considered statistically significant. Statistical analyzes were performed using SPSS version 27 (IBM Corp., Armonk, NY, USA).

### 2.7 Ethical Consideration

Kwame Nkrumah University of Science and Technology Committee on Human Research Publications and Ethics (KNUST‐CHRPE] approved this study with ref: CHRPE/AP/252/24. Study participants were made to know that they have the right to participate or not participate at any stage of the study and that this will not affect the quality of healthcare they were seeking from the study site or elsewhere. Written informed consent was obtained from all participants. All sources of information used in this research was duly acknowledged to avoid any form of plagiarism.

## 3 Results

### 3.1 Demographic Characteristics

About 89 of caregivers responded to the survey questionnaires, the average age of the children with chronic illness under this study was 6.7 ± 2.8 years with minimum and maximum ages of 3 and 14 years respectively. Among 89 caregivers, most children were aged ≥ 6 years (53/89, 59.6%) and male (53/89, 59.6%). The most common conditions were cancer (36/89, 40.4%) and sickle cell disease (30/89, 33.7%) (Table 1). A majority had a history of long admission (64/89, 71.9%). For the caregivers their average was 34.2 ± 6.9 years, with 22 years of minimum and 54 years of maximum. Caregivers were predominantly aged 31–40 years (44/89, 49.4%), female (61/89, 68.5%), and married (78/89, 87.6%). Nearly half had tertiary education (42/89, 47.2%), while 47/89 (52.8%) were unemployed. Most resided in urban areas (49/89, 55.1%), and 51/89 (57.3%) identified as Muslim (Table 1). The average duration of the condition diagnosed was 17.9 ± 24.5 months with shortest duration of 1 month and longest duration of 120 months. The average length of admission stay was 26.9 ± 27.6 days, with minimum and maximum length of admission stay of 2 and 150 days respectively.

**Table 1:** Demographic characteristics of child and caregivers.

		Frequency	Percentage
Child age grouped	Less than 6 years	36	40.4%
	6 years and above	53	59.6%
	Total	89	100.0%
Sex of child	Female	36	40.4%
	Male	53	59.6%
	Total	89	100.0%
Type of chronic condition child is suffering from	Cancer	36	40.4%
	Sickle cell disease	30	33.7%
	Nephrotic syndrome	10	11.2%
	Asthma	7	7.9%
	Cardiac problem	6	6.7%
	Total	89	100.0%
History of long admission because of this chronic condition	Yes	64	71.9%
	No	25	28.1%
	Total	89	100.0%
Caretaker's age grouped	21–30 years	28	31.5%
	31–40 years	44	49.4%
	41–60 years	17	19.1%
	Total	89	100.0%
Sex of caretaker	Female	61	68.5%
	Male	28	31.5%
	Total	89	100.0%
Marital status of caretaker	Married	78	87.6%
	Single	8	9.0%
	Divorced	3	3.4%
	Total	89	100.0%
Caretaker level of education	None	18	20.2%
	Primary	4	4.5%
	Secondary	25	28.1%
	Tertiary	42	47.2%
	Total	89	100.0%
Residence type	Urban	49	55.1%
	Peri‐urban	12	13.5%
	Rural	28	31.5%
	Total	89	100.0%
Occupation of caretaker	Employed	42	47.2%
	Unemployed	47	52.8%
	Total	89	100.0%
Religion of caretaker	Christianity	38	42.7%
	Islam	51	57.3%
	Total	89	100.0%

### 3.2 General Quality of Life and Health Satisfaction Among Caregivers

The average general QoL score of the caregivers was 32.6 ± 24.0, with minimum and maximum scores of 0 and 100 respectively. And their average score for general health satisfaction was 47.5 ± 25.6, minimum score 0 and maximum score 100. Indicating on averages respondents' perception about their overall QoL was poor. While their general satisfaction with heath was moderate. Most (46/89, 51.7%) of the caregivers indicated poor QoL (Table 2).

**Table 2:** Caregivers perceived overall quality of life and health satisfaction.

		Frequency (*n* = 89)	Percentage
How would you rate your quality of life?	Very poor	15	16.9%
	Poor	46	51.7%
	Neither poor nor good	16	18.0%
	Good	10	11.2%
	Very good	2	2.2%
General quality of life score	Mean (SD)	Minimum	Maximum
	32.6 ± 24.0	.0	100.0
How satisfied are you with your health?	Very dissatisfied	4	4.5%
	Dissatisfied	34	38.2%
	Neither satisfied nor dissatisfied	23	25.8%
	Satisfied	23	25.8%
	Very satisfied	5	5.6%
General health satisfaction score	Mean (SD)	Minimum	Maximum
	47.5 ± 25.6	.0	100.0

It is important to note that the general QoL item represents a subjective single‐item perception, whereas the overall QoL score was derived from the mean of the four WHOQOL‐BREF domains. Differences observed between these measures reflect variation between subjective perception and multidimensional domain‐based assessment.

### 3.3 WHOQOL‐BREF Physical Health of Caregivers

The average physical health score from WHOQOL‐BREF analysis was 47.6 ± 17.0, with minimum score of 25 and maximum score of 100. Indicating on averages respondents' rating about their physical health QoL was moderate. From moderate to extreme extent Physical pain prevents majority of the respondents from doing what they want to do. Also, majority of respondents indicated that they need moderates to extreme amount of medical care to function. Again, moderate to extreme extent most of the respondents indicated that they need enough energy for everyday life performance. Positively majority of respondents indicated that they are well able to get around physically. More half of those respondents were not satisfied with their sleep. And less than thirty percent were satisfied with their ability to perform their daily life activities or capacity to work (Table 3).

**Table 3:** Respondents response to WHOQOL‐BREF physical health domain.

		Frequency (*n* = 89)	Percentage
To what extent do you feel that physical pain prevents you from doing what you need to do?	Not at all	8	9.0%
	A little	18	20.2%
	A moderate amount	27	30.3%
	Very much	33	37.1%
	An extreme amount	3	3.4%
How much do you need any medical treatment to function in your daily life?	Not at all	29	32.6%
	A little	12	13.5%
	A moderate amount	14	15.7%
	Very much	29	32.6%
	An extreme amount	5	5.6%
Do you have enough energy for everyday life?	Not at all	6	6.7%
	A little	20	22.5%
	A moderate amount	38	42.7%
	Very much	16	18.0%
	An extreme amount	9	10.1%
How well are you able to get around physically?	Not at all	7	7.9%
	A little	28	31.5%
	A moderate amount	28	31.5%
	Very much	22	24.7%
	An extreme amount	4	4.5%
How satisfied are you with your sleep?	Very dissatisfied	10	11.2%
	Dissatisfied	45	50.6%
	Neither satisfied nor dissatisfied	16	18.0%
	Satisfied	13	14.6%
	Very satisfied	5	5.6%
How satisfied are you with your ability to perform your daily living activities?	Very dissatisfied	3	3.4%
	Dissatisfied	37	41.6%
	Neither satisfied nor dissatisfied	25	28.1%
	Satisfied	20	22.5%
	Very satisfied	4	4.5%
How satisfied are you with your capacity for work	Very dissatisfied	5	5.6%
	Dissatisfied	39	43.8%
	Neither satisfied nor dissatisfied	20	22.5%
	Satisfied	19	21.3%
	Very satisfied	6	6.7%
Physical health score	Mean (SD)	Minimum	Maximum
	47.6 ± 17.0	25.0	100.0

#### 3.3.1 WHOQOL‐BREF Psychological Health Domain Performance by Chronically Ill Child and the Caretaker

The average psychological health score from WHOQOL‐BREF analysis was 50.8 ± 14.7, with minimum and maximum scores of 20.8 and 100. Indicating on averages respondents' rating about their psychological health QoL was moderate. Life enjoyment and meaningful life among respondents dominated from moderate to not at all. Ability to concentrate was moderate 32/89 (36.0%) among the respondents. The ability to accept body appearance dominated the respondent's response from moderate to extreme amount. Most 35/89 (39.3%) of respondents indicated they were Neither satisfied nor dissatisfied with themselves. Seldom to never most 50/89 (56.2%) of respondents indicated experiencing negative feeling such as blue mood, despair, anxiety, or depression (Table 4).

**Table 4:** Respondents response to WHOQOL‐BREF psychological health domain.

		Frequency (*n* = 89)	Percentage
How much do you enjoy life?	Not at all	11	12.4%
	A little	34	38.2%
	A moderate amount	28	31.5%
	Very much	11	12.4%
	An extreme amount	5	5.6%
To what extent do you feel your life to be meaningful?	Not at all	7	7.9%
	A little	27	30.3%
	A moderate amount	31	34.8%
	Very much	18	20.2%
	An extreme amount	6	6.7%
How well are you able to concentrate?	Not at all	6	6.7%
	A little	26	29.2%
	A moderate amount	32	36.0%
	Very much	15	16.9%
	An extreme amount	10	11.2%
Are you able to accept your body's appearance?	Not at all	1	1.1%
	A little	20	22.5%
	A moderate amount	35	39.3%
	Very much	28	31.5%
	An extreme amount	5	5.6%
How satisfied are you with yourself?	Very dissatisfied	3	3.4%
	Dissatisfied	25	28.1%
	Neither satisfied nor dissatisfied	35	39.3%
	Satisfied	19	21.3%
	Very satisfied	7	7.9%
How often do you have negative feelings such as blue mood, despair, anxiety, or depression?	Never	18	20.2%
	Seldom	32	36.0%
	Quit often	23	25.8%
	Very often	13	14.6%
	Always	3	3.4%
Psychological health score	Mean (SD)	Minimum	Maximum
	50.8 ± 14.7	20.8	100.0

#### 3.3.2 WHOQOL‐BREF Social Relationship Domain Performance by Chronically Ill Child and the Caretaker

The average social relationship score from WHOQOL‐BREF analysis was 53.0 ± 19.1, with minimum and maximum scores of 16.7 and 100 respectively. Indicating on averages respondents' rating about their social relationship QoL was moderate. Less than forty percent were satisfy with their relation. Also, about 21/89 (23.6%) of the respondents were satisfied with their sex life. However, significant number were satisfied with the support they get from friends (Table 5).

**Table 5:** Respondents response to WHOQOL‐BREF social relationship domain.

		Frequency (*n* = 89)	Percentage
How satisfied are you with your relationships?	Very dissatisfied	2	2.2%
	Dissatisfied	19	21.3%
	Neither satisfied nor dissatisfied	34	38.2%
	Satisfied	27	30.3%
	Very satisfied	7	7.9%
How satisfied are you with your sex life?	Very dissatisfied	13	14.6%
	Dissatisfied	30	33.7%
	Neither satisfied nor dissatisfied	25	28.1%
	Satisfied	15	16.9%
	Very satisfied	6	6.7%
How satisfied are you with the support you get from your friends?	Very dissatisfied	4	4.5%
	Dissatisfied	10	11.2%
	Neither satisfied nor dissatisfied	23	25.8%
	Satisfied	43	48.3%
	Very satisfied	9	10.1%
Social relationship score	Mean (SD)	Minimum	Maximum
	53.0 ± 19.1	16.7	100.0

#### 3.3.3 WHOQOL‐BREF Environmental Domain Performance by Chronically Ill Child and the Caretaker

The average environmental domain score from WHOQOL‐BREF analysis was 47.9 ± 14.4, with minimum and maximum scores of 18.8 and 96.9 respectively. Indicating on averages respondents' rating about their environmental QoL was moderate. Generally, the respondents indicated they feel safe in their daily lives from moderate to extreme amount. And rating healthiness of their environment from moderate to extreme amount. However, majority 53/89 (59.5%) indicated little to no amount of available money to meet their needs. Most of them indicated moderated to extreme availability of information for their daily needs. The opportunity for leisure activities was little to no at all for most the respondents. Only 44/89 (49.5%) of respondents the respondents indicated they were satisfied with their place of living. About 32/89 (36.0%) of respondents the indicated they were satisfied with the health services access. Finally, transport satisfaction was by only 25/89 (28.1%) of the respondents (Table 6).

**Table 6:** Respondents' response to WHOQOL‐BREF environment domain.

		Frequency (*n* = 89)	Percentage
How safe do you feel in your daily life?	Not at all	5	5.6%
	A little	22	24.7%
	A moderate amount	33	37.1%
	Very much	25	28.1%
	An extreme amount	4	4.5%
How healthy is your physical environment?	Not at all	2	2.2%
	A little	16	18.0%
	A moderate amount	36	40.4%
	Very much	28	31.5%
	An extreme amount	7	7.9%
Have you enough money to meet your needs?	Not at all	13	14.6%
	A little	40	44.9%
	A moderate amount	29	32.6%
	Very much	6	6.7%
	An extreme amount	1	1.1%
How available to you is the information you need in your day‐to‐day life?	Not at all	3	3.4%
	A little	34	38.2%
	A moderate amount	19	21.3%
	Very much	31	34.8%
	An extreme amount	2	2.2%
To what extent do you have the opportunity for leisure activities?	Not at all	11	12.4%
	A little	39	43.8%
	A moderate amount	27	30.3%
	Very much	11	12.4%
	An extreme amount	1	1.1%
How satisfied are you with the conditions of your living place?	Very dissatisfied	3	3.4%
	Dissatisfied	21	23.6%
	Neither satisfied nor dissatisfied	21	23.6%
	Satisfied	33	37.1%
	Very satisfied	11	12.4%
How satisfied are you with your access to health services?	Very dissatisfied	2	2.2%
	Dissatisfied	23	25.8%
	Neither satisfied nor dissatisfied	32	36.0%
	Satisfied	21	23.6%
	Very satisfied	11	12.4%
How satisfied are you with your transport?	Very dissatisfied	8	9.0%
	Dissatisfied	28	31.5%
	Neither satisfied nor dissatisfied	28	31.5%
	Satisfied	21	23.6%
	Very satisfied	4	4.5%
Environmental score	Mean (SD)	Minimum	Maximum
	47.9 ± 14.4	18.8	96.9

### 3.4 Overall Quality of Life Among Chronic Ill Children's Caregivers

The overall QoL scores was achieved by the mean of the four domain scores. The mean value for the overall scores was 49.9 ± 13.6, with minimum score of 31 and maximum score of 98. For regression analysis, QoL was dichotomized into poor (≤ 45) and not poor (> 45) to facilitate binary logistic regression. However, this approach may reduce variability and should be interpreted with caution.

#### 3.4.1 Factors Associated With Quality of Life Among Care Takers of Chronically Ill Children

##### 3.4.1.1 Chi‐Quare Analysis for Factors Associated With Quality of Life Among Care Takers of Chronically Ill Children

Chi‐square analysis showed clear differences in caregivers' QoL across several characteristics. Caregivers of children with cancer had a much higher proportion of poor QoL (27/36, 75.0%) compared with those caring for children with sickle cell disease (5/30, 16.7%) and other chronic conditions (12/23, 52.2%), indicating a strong association between type of chronic disease and caregiver QoL (χ^2^ = 22.368, *p* < 0.001).

A history of prolonged hospital admission was also associated with poorer QoL, with 38/64 (59.4%) of caregivers reporting poor QoL compared to 6/25 (24.0%) among those without such history (χ^2^ = 9.000, *p* = 0.003).

Lower educational attainment was associated with worse outcomes; 29/47 (61.7%) of caregivers with below tertiary education reported poor QoL compared with 15/42 (35.7%) among those with tertiary education (χ^2^ = 5.993, *p* = 0.014). Similarly, unemployment was linked with poorer QoL (30/47, 63.8%) compared to employed caregivers (14/42, 33.3%) (χ^2^ = 8.252, *p* = 0.004).

Religious affiliation also showed variation, with 24/38 (63.2%) of Christian caregivers reporting poor QoL compared to 20/51 (39.2%) among Muslim caregivers (χ^2^ = 4.994, *p* = 0.004).

In contrast, no meaningful differences were observed for child age (χ^2^ = 0.905, *p* = 0.341), child sex (χ^2^ = 0.905, *p* = 0.341), caregiver age group (χ^2^ = 2.152, *p* = 0.341), caregiver sex (χ^2^ = 2.078, *p* = 0.149), marital status (χ^2^ = 1.027, *p* = 0.598), or residence type (χ^2^ = 3.928, *p* = 0.140) (Table 7).

**Table 7:** Chi‐square analysis for factors associated with quality of life among caregivers.

		Overall quality of life				Test Statistics
		Poor quality of life		Good quality of life		
Child age grouped	Less Than 6 years	20	55.6%	16	44.4%	*X* ^2^ = 0.905
	6 years and above	24	45.3%	29	54.7%	*p* = 0.341
Sex of child	Female	20	55.6%	16	44.4%	*X* ^2^ = 0.905
	Male	24	45.3%	29	54.7%	*p* = 0.341
Type of chronic disease	Cancer	27	75.0%	9	25.0%	***X*** ^**2**^ = **22.368**
	Sickle cell disease	5	16.7%	25	83.3%	***p*** < **0.001**
	Other	12	52.2%	11	47.8%	
History of long admission because of this chronic condition	Yes	38	59.4%	26	40.6%	***X*** ^**2**^ = **9.000**
	No	6	24.0%	19	76.0%	***p*** = **0.003**
Caretaker's age grouped	21–30 years	17	60.7%	11	39.3%	*X* ^2^ = 2.152
	31–40 years	19	43.2%	25	56.8%	*p* = 0.341
	41–60 years	8	47.1%	9	52.9%	
Sex of caretaker	Female	27	44.3%	34	55.7%	***X*** ^**2**^ = **2.078**
	Male	17	60.7%	11	39.3%	***p*** = **0.149**
Marital status of caretaker	Married	37	47.4%	41	52.6%	*X* ^2^ = 1.027
	Single	5	62.5%	3	37.5%	*p* = 0.598
	Divorced	2	66.7%	1	33.3%	
Caretaker educational level	Below tertiary	29	61.7%	18	38.3%	***X*** ^**2**^ = **5.993**
	Tertiary	15	35.7%	27	64.3%	***p*** = **0.014**
Residence type	Urban	20	40.8%	29	59.2%	***X*** ^**2**^ = **3.928**
	Peri‐urban	6	50.0%	6	50.0%	***p*** = **0.140**
	Rural	18	64.3%	10	35.7%	
Occupation of caretaker	Employed	14	33.3%	28	66.7%	***X*** ^**2**^ = **8.252**
	Unemployed	30	63.8%	17	36.2%	***p*** = **0.004**
Religion of caretaker	Christianity	24	63.2%	14	36.8%	***X*** ^**2**^ = **4.994**
	Islam	20	39.2%	31	60.8%	***p*** = **0.004**

##### 3.4.1.2 Correlation Between Duration of Child Diagnosed With Chronic Condition, Duration of Long Admission and Overall Quality of Life Among Care Takers

In the correlation analysis the overall QoL had significant association with duration since the child was diagnosed with this chronic condition with mild positive coefficient, rho (89) = 0.3, *p* = 0.001. and on the other had mild negative correlation with the duration of long admission, rho (89) = −0.3, *p* = 0.004 (Table 8). Spearman's correlation was used due to non‐normal distribution of variables.

**Table 8:** Correlation between duration of child diagnosed with chronic condition, duration of long admission and overall quality of life among care takers.

			Duration since your child was diagnosed with this chronic condition (months)	Duration of this long admission (In days)
Spearman's rho	Duration since your child was diagnosed with this chronic condition (months)	Correlation coefficient	—	
		Sig. (2‐tailed)		
		*N*	89	
	Duration of this long admission (In days)	Correlation coefficient	0.070	—
		Sig. (2‐tailed)	0.517	
		*N*	89	89
	Overall QoL	Correlation coefficient	0.343**	−0.300**
		Sig. (2‐tailed)	0.001	0.004
		*N*	89	89

** Correlation is significant at the 0.01 level (2‐tailed).

##### 3.4.1.3 Multiple Variable Analysis for Factors Predicting Overall Quality of Life Among Care Takers of Children With Chronic Conditions

In the logistic regression analysis involving multiple variables, care takers who were unemployed were less likely to have good QoL as compared to those employed with the odds of 0.3 (AOR = 0.27, 95%, C.I. = 0.083–0.882). However, those who were of the Islamic religion were 3.1 times more likely to experience good QoL as compared to those of the Christianity faith (AOR = 4.05, 95%, C.I. = 1.241–13.240). Finally, care takers of children diagnosed with sickle cell disease were 6.8 times more likely to experience good QoL when compared to care takers of children diagnosed with cancers (AOR = 7.75, 95%, C.I. = 1.228–48.901) (Table 9).

**Table 9:** Binary logistics regression for factors associated with overall quality of life among study participants.

		B	Sig.	AOR	95% C.I. for AOR	
					Lower	Upper
Type of chronic disease	Cancer		Ref			
	Sickle cell disease	2.048	0.029	7.750	1.228	48.901
	Other	0.560	0.450	1.752	0.410	7.489
History of long admission because of this chronic condition	Yes		Ref			
	No	0.602	0.411	1.825	0.434	7.668
Sex of caretaker	Female		Ref			
	Male	−0.455	0.451	0.635	0.195	2.071
Caretaker educational level	Below tertiary		Ref			
	Tertiary	0.108	0.874	1.114	0.291	4.259
Residence type	Urban		0.872			
	Peri‐urban	−0.228	0.791	0.796	0.147	4.316
	Rural	0.239	0.726	1.270	0.334	4.829
Occupation of caretaker	Employed					
	Unemployed	−1.308	0.030	0.270	0.083	0.882
Religion of caretaker	Christianity					
	Islam	1.400	0.020	4.054	1.241	13.240
Duration since your child was diagnosed with this chronic condition (months)		0.011	0.528	1.011	0.977	1.046
Duration of this long admission (In days)		−0.003	0.790	0.997	0.972	1.022
Constant		−1.080	0.275	0.340		

Given the relatively small sample size (*n* = 89), the number of variables included in the multivariable logistic regression model was carefully limited. However, the possibility of overfitting cannot be excluded, and results should be interpreted with caution.

The wide confidence intervals observed in some estimates (e.g., sickle cell disease) suggest limited precision, likely due to the small sample size, and indicate that these findings should be interpreted cautiously.

## 4 Discussion

QoL is a patient‐reported outcome measure (PROM) that is becoming more and more important in cost‐effectiveness studies, clinical practice, and outcome evaluation in clinical trials. HRQoL is a key component of QoL [16]. In this current study on average respondents' perception about their overall QoL was poor. While their general satisfaction with heath was moderate. This is like earlier reports as evidence from the literature points to a significant decline in parental QoL [17, 18].

QoL is a measure of health that encompasses social, psychological, and physical aspects and whose essence is not fully reflected by traditional clinical tools [16].

Parents with chronic ill children typically spend a lot of time raising their kids, which can lead to isolation, negatively damage their careers, and restrict their free time [18]. Children's chronic illness suggests redefining roles and responsibilities as well as restructuring family dynamics two difficult things to do [18]. Parents who are unable to handle these difficulties well sometimes lament their psychological discomfort, worry, and melancholy [18, 19].

The findings of this study suggest that the average respondents' rating of their psychological health QoL was moderate for average level of well‐being. Areas of concern were the struggle to find purpose and happiness, difficulty with focus and mental clarity, and low self‐esteem. Most respondents were neither satisfied nor dissatisfied with themselves. Fortunately, most were seldom or never experiencing negative feelings like blue mood, despair, anxiety, or depression. This study finding indicates caregivers of children with chronic in Ghana may be experiencing some mental health challenges, which could impact their overall quality of life [20]. This emphasis the importance of addressing psychological health and well‐being of all [21].

On the social QoL, the findings of this study suggest average level of satisfaction. Few were satisfied with their romantic relationships and sex life, indicating a potential area of concern [22]. The dissatisfaction with romantic relationships and sex life is also consistent with previous research, which has identified relationship issues as a common concern [23]. A significant number of respondents were satisfied with the support they receive from friends, highlighting the importance of social support networks [24]. These findings are consistent with previous research, which has shown that social relationships are a crucial aspect of overall QoL [25]. Satisfaction with friend support highlights the importance of social support in maintaining good mental health [26]. The moderate rating suggests that parents or caregivers of chronic ill children may be experiencing some challenges in their social relationships, which could impact their overall well‐being [27].

On the physical QoL, the findings suggest average level of physical well‐being among caregivers of children with chronic conditions. Areas of concern were physical pain and dependency on medical care to function, low energy levels, low sleep satisfaction and low satisfaction with ability to perform daily life activities or capacity to work. These findings align with previous research highlighting the importance of addressing physical health and functional ability [28]. The moderate rating suggests that respondents may be experiencing some physical health challenges, which could impact their overall QoL [29].

This study findings suggested average level of satisfaction with living environment among caregivers of chronic ill children. They felt safe with their daily lives, with environmental healthiness. However, most caregivers reported having little to no money to meet their daily needs, and little to no opportunity for leisure activities for relaxation and enjoyment. On satisfaction with living conditions caregivers are not satisfied with their place of living, health services access, and transportation, indicating room for improvement in these areas. These findings align with previous research highlighting the importance of environmental factors in QoL. The moderate rating suggests that respondents may be experiencing some environmental challenges, which could impact their overall well‐being [30].

The study found that, on average, respondents reported a good QoL. The analysis identified three significant factors associated with overall good QoL among caregivers: employment status, religious affiliation and medical diagnosis. Caregivers who were employed were more likely to report a good QoL compared to those who were unemployed. And this is in‐line with literature [31, 32]. This suggests that employment provides a sense of purpose, financial stability, and social support, which are essential for overall well‐being.

Research has indicated that there is a positive correlation between religious characteristics and feelings of happiness and life satisfaction. There are very few research that show a negative correlation between subjective well‐being and religiosity [33, 34]. Also, in this present study caregivers who identified as Muslim were more likely to report a good QoL compared to those who identified as Christian. This may be attributed to the strong social support and sense of community often found in Muslim communities [35, 36]. The observed association between religious affiliation and QoL should be interpreted with caution, as it may reflect underlying unmeasured factors such as social support systems, socioeconomic status, or cultural coping mechanisms rather than a direct effect of religion itself.

Finally, caregivers of children with sickle cell disease were more likely to report a good QoL compared to those caring for children with cancer. This may be due to the differing levels of financial stress, uncertainty, and emotional burden associated with each condition. Both cancer and sickle cell disease (SCD) can be expensive to treat in Africa, but the cost of treatment varies widely depending on the specific type of cancer, the severity of SCD, and the country or region. However, in general, cancer treatment is often more expensive than SCD treatment in Africa. And Increased life expectancy, decreased infant mortality rates, and enhanced general well‐being, financial quality of life, and health are all linked to greater income levels [37].

These findings highlight the importance of addressing the practical, social, and emotional needs of caregivers to support their overall QoL. Interventions aimed at promoting employment opportunities, social support, and religious coping may be beneficial for caregivers. However, this study has several limitations. First, it was conducted at a single tertiary facility, which may limit the generalizability of the findings to other settings. Second, the relatively small sample size reduces statistical power and may contribute to imprecise estimates, as reflected in the wide confidence intervals observed. Third, the cross‐sectional design precludes causal inference between variables. Fourth, data were self‐reported and may be subject to recall and social desirability bias. Additionally, the grouping of heterogeneous conditions under the “other” category (e.g., asthma, nephrotic syndrome, and cardiac conditions) may have masked important differences between specific disease types and should be interpreted with caution. Finally, potential confounding variables such as socioeconomic status and family support systems were not fully assessed.

## 5 Conclusion

The study found that the overall QoL among caregivers of chronically ill children was moderate and significantly associated with socio‐demographic and illness‐related factors. Employment status, religious affiliation, and the type of chronic illness significantly predicted caregiver QoL. Targeted support interventions, particularly for unemployed caregivers and those managing children with more severe conditions like cancer, are essential to improve their well‐being.

## Author Contributions

**Rhubamatu Iddrisu:** conceptualization, investigation, funding acquisition, writing—original draft, writing—review and editing, visualization, methodology, validation, project administration, software, data curation, supervision, resources. **Abdul Rauf Alhassan:** conceptualization, investigation, funding acquisition, writing—original draft, writing—review and editing, visualization, validation, methodology, software, formal analysis, project administration, resources, supervision, data curation. **Mariam Kaleem‐Ahmed:** funding acquisition, writing—original draft, software, project administration, resources, data curation, supervision, methodology, validation, visualization, writing—review and editing, conceptualization, investigation. **Yaa Nyarko Adjeso:** conceptualization, investigation, funding acquisition, writing—original draft, visualization, validation, methodology, writing—review and editing, software, project administration, resources, supervision, data curation.

## Funding

The authors have nothing to report.

## Disclosure

All authors have read and approved the final version of the manuscript. Abdul Rauf Alhassan had full access to all of the data in this study and takes complete responsibility for the integrity of the data and the accuracy of the data analysis.

## Conflicts of Interest

The authors declare no conflicts of interest.

## Transparency Statement

Abdul Rauf Alhassan affirms that this article is an honest, accurate, and transparent account of the study being reported; that no important aspects have been omitted; and that any discrepancies from the planned study have been explained.

## Data Availability

Data for the findings of this study are available from the corresponding author upon request.
